# New Dystrophin/Dystroglycan interactors control neuron behavior in *Drosophila *eye

**DOI:** 10.1186/1471-2202-12-93

**Published:** 2011-09-26

**Authors:** April K Marrone, Mariya M Kucherenko, Valentyna M Rishko, Halyna R Shcherbata

**Affiliations:** 1 Max Planck Institute for biophysical chemistry, Research group of Gene Expression and Signaling, Am Fassberg 11, 37077, Goettingen, Germany; 2 Ivan Franko National University of Lviv, Department of Genetics and Biotechnology, Hrushevsky 4, 79005, Ukraine

## Abstract

**Background:**

The Dystrophin Glycoprotein Complex (DGC) is a large multi-component complex that is well known for its function in muscle tissue. When the main components of the DGC, Dystrophin (Dys) and Dystroglycan (Dg) are affected cognitive impairment and mental retardation in addition to muscle degeneration can occur. Previously we performed an array of genetic screens using a *Drosophila *model for muscular dystrophy in order to find novel DGC interactors aiming to elucidate the signaling role(s) in which the complex is involved. Since the function of the DGC in the brain and nervous system has not been fully defined, we have here continued to analyze the DGC modifiers' function in the developing *Drosophila *brain and eye.

**Results:**

Given that disruption of *Dys *and *Dg *leads to improper photoreceptor axon projections into the lamina and eye neuron elongation defects during development, we have determined the function of previously screened components and their genetic interaction with the DGC in this tissue. Our study first found that mutations in *chif, CG34400, Nrk*, *Lis1, capt *and *Cam *cause improper axon path-finding and loss of *SP2353, Grh, Nrk, capt, CG34400, vimar, Lis1 *and *Cam *cause shortened rhabdomere lengths. We determined that *Nrk*, *mbl*, *capt *and *Cam *genetically interact with *Dys *and/or *Dg *in these processes. It is notable that most of the neuronal DGC interacting components encountered are involved in regulation of actin dynamics.

**Conclusions:**

Our data indicate possible DGC involvement in the process of cytoskeletal remodeling in neurons. The identification of new components that interact with the DGC not only helps to dissect the mechanism of axon guidance and eye neuron differentiation but also provides a great opportunity for understanding the signaling mechanisms by which the cell surface receptor Dg communicates via Dys with the actin cytoskeleton.

## Background

Muscular dystrophies (MDs) are a group of diseases that are characterized by progressive muscular degeneration and concomitant loss of muscular strength ultimately leading to skeletal muscle deterioration and cardiac and/or respiratory failure [1-3]. In addition, MDs are often associated with brain defects. Based upon the clinical symptoms of MDs they are categorized into various subtypes and currently no cures or preventions exist for these diseases, making them a worthwhile field of research. The most severe form of MD is Duchenne MD (DMD), an X-linked fatal disorder that afflicts approximately 1 out of every 3,500 males worldwide. The DMD pathology contains a subset of individuals (about 1 in 3) that suffer from cognitive impairment and mental retardation, and these attributes of the disease appear to be independent from the muscular handicap [4,5].

DMD arises from the loss of the Dystrophin (Dys) protein product, which provides a link between cytoskeletal actin and the ECM via the glycoprotein Dystroglycan (Dg). Dys binds Dg along with several other transmembrane proteins (two syntrophins, two dystrobrevins, and four sarcoglycans) [6,7] to assemble the Dystrophin Glycoprotein Complex (DGC).

Mutations of Dystroglycan, the key transmembrane component of the DGC, lead to discontinuities in the basement membrane surrounding the cerebral cortex and disorganized cortical layering (for review see [8]). In addition, Dg hypoglycosylation leads to congenital muscular dystrophies (CMDs), of which some feature brain defects including cobblestone (type II) lissencephaly. This type of lissencephaly is characterized by heterotopic glia and neurons that disrupt the laminar organization of the cerebral cortex [9], and mutations in glycosyltransferases that act upon α-Dg have been linked to these disorders [10-18].

Disruption of the DGC not only affects cerebral cortex layering and lamina organization but also leads to physiological defects in neuron function. DMD patients and *mdx*^Cv3 ^(DMD mouse model missing the 427 and 70 kD isoforms of Dys) and Dystroglycan knockout mice have reduced b-wave amplitudes in electroretinograms [19-22] supporting its specific role in the nervous system establishment and function. While the role of the DGC in muscle has been intensively studied, its function in the brain and nervous system has not been completely defined.

*Drosophila *has been demonstrated to serve as a useful model for studying the DGC *in vivo *since DGC mutants develop symptoms similar to MD patients [23-25]. Key human and *Drosophila *DGC components are evolutionarily conserved and interact in a similar manner [24,26,27]. As in mammals, in *Drosophila *proteins of the DGC are not only found at the muscle sarcolemma but also at the neuromuscular junction and in the PNS and CNS [23-25,28-32]. In the *Drosophila *brain Dg is expressed in R cells, glia and neurons, indicating that this protein has an important role in nervous system function [24,33,34]. *Drosophila *R cells provide an excellent system in which to study axon guidance, growth and elongation, negative and attractive guidance cues and neuron polarity establishment that later results in cell shape rearrangement. Both Dys and Dg affect photoreceptor cell elongation and are required in neurons and glia for proper photoreceptor axon migration [24,33]. Based on these phenotypes we performed a genetic interaction screen in order to find novel neuronal DGC components. We analyzed potential interactors that were identified in a previous screen to interact with Dys and/or Dg in muscle degeneration [35] and have found new components that interact with primarily Dys and to a lesser extent Dg in developing eye neurons. Among them are the calcium binding protein Calmodulin (Cam), Neurospecific receptor kinase (Nrk), a splicing factor Muscleblind (Mbl) and the actin recycling protein Capulet (Capt). Since most of these proteins have been shown to affect actin organization, polymerization and recycling, these results suggest that in neurons the DGC is involved in the processes of actin cytoskeleton regulation.

## Results

### Dys and Dg are expressed in Drosophila larval and adult nervous system

**Figure 1:**
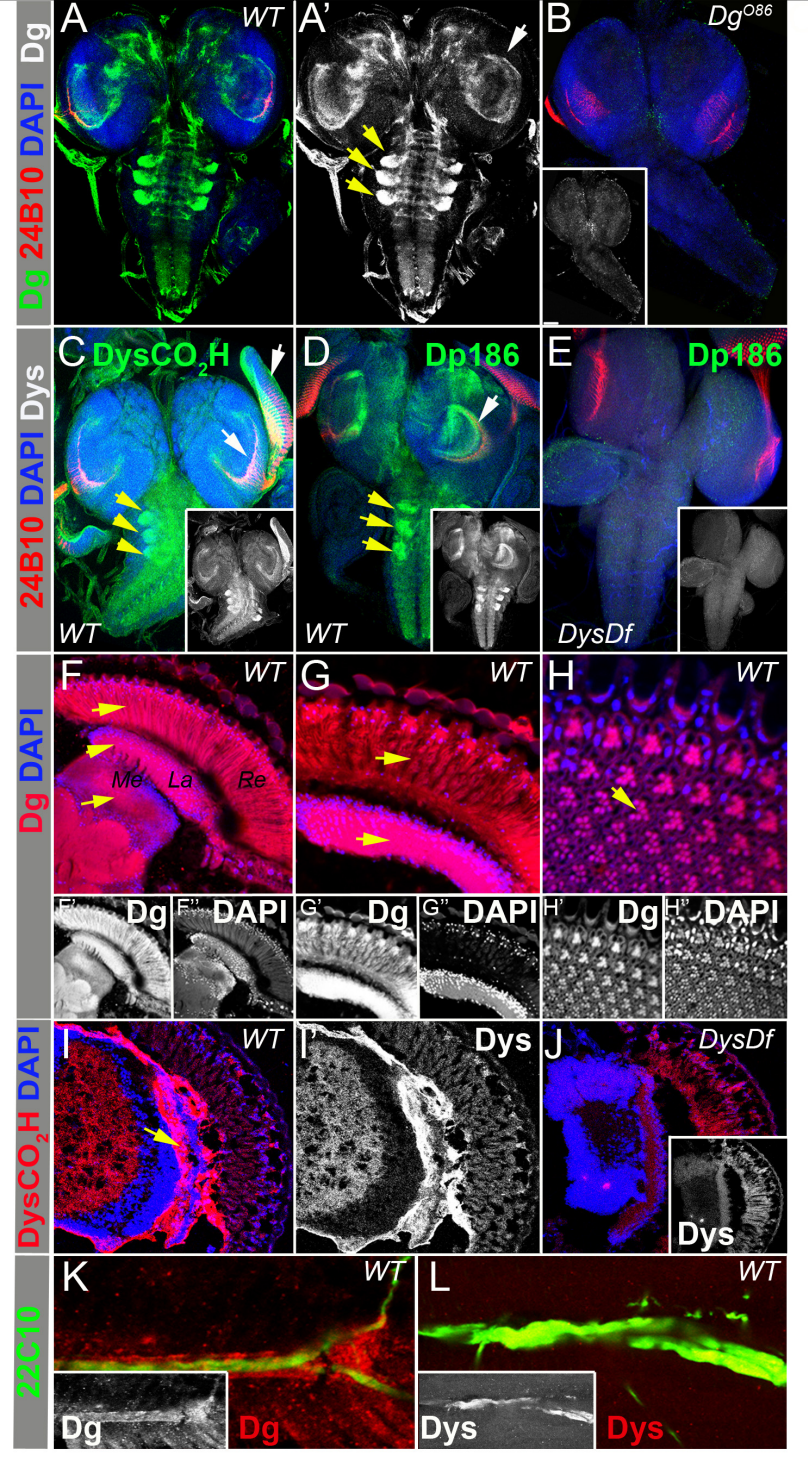
**Dys and Dg are expressed in Drosophila larval and adult nervous systems**. Expression pattern for Dg (A-B) and Dys (C-E) in *Drosophila *3rd instar larval brains. Photoreceptor neurons are marked with the 24B10 antibody (red). The Dys antibody that recognizes all Dys isoforms by targeting the carboxy terminus shows localization in the brain and the eye discs (C) whereas an antibody that recognizes the Dp186 isoform is present only in the brain (D). Dg (B) and Dys (E) staining is absent in *Dg *and *Dys *loss-of-function homozygous mutants respectively. White arrows indicate Dys and Dg signal in the optic lobes and yellow arrows show staining in clusters at the lateral sides of the neuropile. The expression pattern for Dg (F-H) and Dys (I) in the adult brain. Arrows show strong Dg and Dys signals detected in the lamina *(La) *and medulla *(Me) *and Dg expression is also seen in the retina *(Re)*. Dys staining is diminished from the brain of *Dys *loss-of-function homozygous mutant (J). Expression pattern for Dg (K) and Dys (L) in *Drosophila *motorneurons marked with the 22C10 antibody (green).

In this study we used antibodies that specifically recognize Dys and Dg proteins in order to detect the expression pattern of the main DGC components in the larval and adult nervous system. Previously it has been shown that Dg is expressed in neurons and glia in the larval *Drosophila *brain; high levels of Dg were detected in axons of photoreceptor sensory neurons, in the optic stalk, and in glial cells in the optic lobes [24]. Now we show that Dg is also expressed in the neuropile of 3^rd ^instar larval CNS and in three symmetric clusters at the lateral sides of the neuropile (Figure 1A). As expected, we observed a similar expression pattern for Dg's binding partner Dys (Figure 1C-D). The carboxy terminal specific Dys antibody that recognizes all isoforms [28], localizes in the neuropile, the optic lobes and in the axons of photoreceptor sensory neurons (Figure 1C). In accord with previously reported data [30], a strong Dys signal was detected in the neuropile and in the optic lobes when using an antibody that recognizes the CNS-specific Dp186 isoform (Figure 1D). In the adult *Drosophila *brain Dg is detected in the medulla and the lamina (Figure 1F-G) and a strong Dg signal is also seen in the retina (Figure 1F-). Dys expression in the adult brain appears to be localized to the lamina (Figure 1I). Dys and Dg are not only expressed in the CNS, but also the PNS (Figure 1K-L): Dg staining was seen in and around the motoneurons (Figure 1K), while the Dys signal was overlaid with neuronal 22C10 staining (Figure 1L). In *Dg *and *Dys *loss-of-function homozygous mutants (*Dg*^*O86 *^and *DysDf*) Dg and Dys signals were diminished (Figure 1B, E, J) implying that the detected expression pattern was specific in accord with previous reports for these antibodies [24,28,35]. A definite expression pattern for Dys and Dg in the *Drosophila *nervous system implies that the DGC is involved in nervous system development. As has been shown previously, disruption of Dys or Dg gives rise to a disorganized lamina plexus, and these two components genetically interact [24]. This abnormal photoreceptor guidance can result from growth cone malfunction, compromised cell polarity, malformed actin cytoskeleton or disrupted glia-neuron communication. During *Drosophila *development, eye discs contain ommatidia and photoreceptor neurons that project axons down the optic stalk to innervate the brain. For each ommatidia there are eight photoreceptors (R-cells), where six of the eight cells stop at the superficial lamina forming the lamina plexus, and the other two photoreceptors project further into the medulla. Later, during pupation photoreceptors differentiate and undergo morphological changes including elongation and Dys and Dg both affect this process [24,33]. Since recently we identified genes that interact with Dys and/or Dg in age related muscle degeneration [35], in the present work we analyzed these gene candidates for their involvement together with the DGC in neuron behavior during *Drosophila *visual system development.

### Muscle DGC-interacting components also affect photoreceptor axon guidance and rhabdomere length

**Figure 2:**
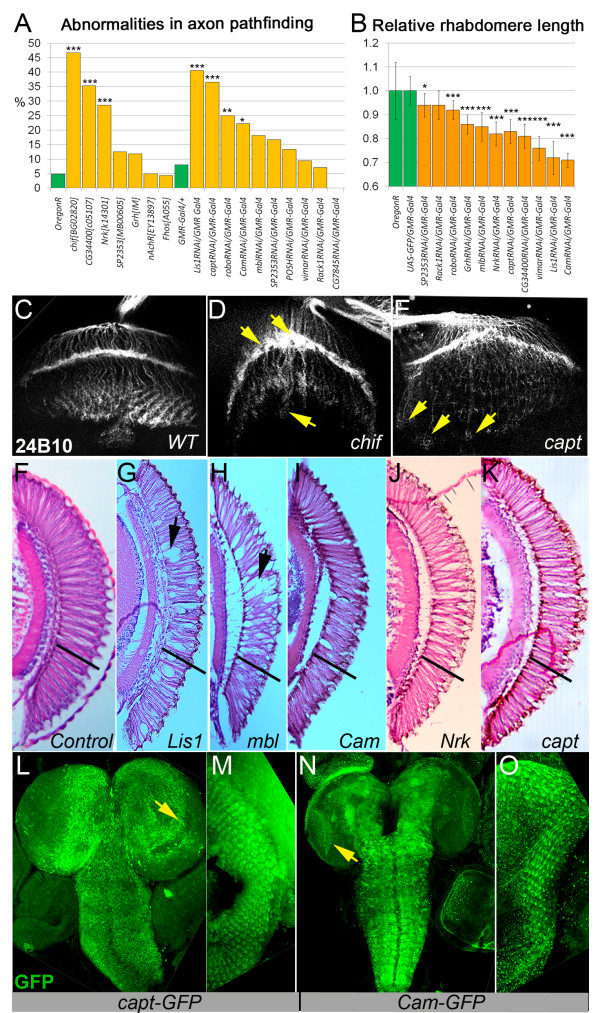
**Requirement of screened components for photoreceptor cell development**. (A) Bar graph represents frequency of defective photoreceptor axon projections in 3^rd ^instar larval brain. Statistics were determined using the χ^2^-test with Yate's correction where ***p ≤ 0.001, **p ≤ 0.01, *p ≤ 0.05. (B) Bar graph shows relative rhabdomere length in adult flies with down regulation of tested components. Statistics were determined using a one-tailed Student's t-test where ***p ≤ 0.001, **p ≤ 0.01, *p ≤ 0.05. (C) Photoreceptor axons projection in wild type 3^rd ^instar larval brain visualized with the 24B10 antibody. (D-E) Abnormal R cells growth and termination in 3^rd ^instar larvae with down regulated Chif and Capt (*chif*^*BG02820*^*, capt*^*RNAi*^*/GMR-Gal4*). Arrows indicate irregular axon termination in the lamina plexus, lamina plexus breaks and sites of overgrown axons. (F-K) Exemplary brain sections represent rhabdomere length and morphology in tested mutants in comparison to control (F). Arrows show vacuoles in retina and black bars indicate *wt *rhabdomere length. (L-O) *capt *and *Cam *GFP trap lines show expression patterns for Cam and Capt in the larval brain. (M, O) Enlarged view of eye discs. Arrows indicate expression pattern in optic lobes.

First, we questioned if previously identified DGC-interacting components have a role in neuronal architecture. Analysis of the 24B10 staining pattern in 3^rd ^instar larvae found a significantly increased frequency of axon migration abnormalities in the brains of seven of the sixteen analyzed mutants (Calmodulin (*Cam*), Capulet (*capt*), Neurospecefic receptor kinase (*Nrk*), Chiffon (*chif*), *CG34400*, Lissencephaly-1 (*Lis1*) and Roundabout (*robo*)) - 22-47% in comparison to less than 10% observed in control animals (Figure 2A, Table 1), resulting in lamina plexus breaks or overgrown axons (Figure 2D-E). Next we asked if the DGC interactors are required to provide proper photoreceptor differentiation via analysis of adult brain histological sections. We used *RNAi *transgenic mutants crossed to *GMR-Gal4 *to target gene expression specifically in the visual system and identified shorter rhabdomeres in *Cam, capt, Nrk, mbl, CG34400, Lis1*, Visceral mesodermal armadillo repeats (*vimar*)*, SP2353 *and Grainyhead (*Grh*) (Figure 2B, F-K, Table 1). Interestingly, we also noticed vacuoles in the retinas of *Mbl*^*RNAi*^*/GMR-Gal4 *and *Lis1*^*RNAi*^*/GMR-Gal4 *mutants (Figure 2G-H).

**Table 1:** Genetic interactors of *Dys *and *Dg *in muscle have photoreceptor axon pathfinding defects and shortened rhabdomeres

Genotype	Defective lamina plexuses (%)	n, analyzed optic lobes	**χ**^**2**^**-value**	Relative rhabdomere length	n, analyzed eyes	p-value
*OregonR *(Control)	4.9	41		1.0 ± 0.12	8	-

*CG34400[c05107]*	**35.3**	**17**	21.7 ***	-	-	-

*chif[BG02820]*	**46.7**	**15**	28.4 ***	-	-	-

*Fhos[A055]*	4.3	23	3.3 × 10^-2^	-	-	-

*Grh[IM]*	11.8	17	2.1	-	-	-

*nAcRα-30D[EY13897]*	5.0	40	6.5 × 10^-2^	-	-	-

*Nrk[k14301]*^*1*^	**28.6**	**14**	15.5 ***	-	-	-

*SP2353[MB00605]*	12.5	32	2.6	-	-	-

*eyeFlpFRT/Lis1FRT*	-	-	-	0.94 ± 0.08	10	0.20

*GMR-Gal4/+*	8.0	25	-	1.00 ± 0.06	32	-

*Cam*^*RNAi*^*/GMR-Gal4*	**22.2**	**18**	5.8*	**0.71 ± 0.03**	**6**	**1.3 × 10**^-12^***

^*2*^*capt*^*RNAi*^*/GMR-Gal4*	**36.6**	**71**	17.1 ***	**0.81 ± 0.03**	**18**	**2.9 × 10**^-11^***

^*3*^*capt*^*RNAi*^*/GMR-Gal4*	**42.9**	**49**	22.6***	**0.76 ± 0.07**	**18**	**1.0 × 10**^-14^***

*CG7845*^*RNAi*^*/GMR-Gal4*	0.0	13	6.1	-	-	-

*Fkbp13*^*RNAi*^*/GMR-Gal4*	4.7	21	6.1	-	-	-

*Lis1*^*RNAi*^*/GMR-Gal4*	**40.6**	**32**	20.5 ***	**0.72 ± 0.07**	**18**	**1.8 × 10**^-11^***

*mbl*^*RNAi*^*/GMR-Gal4*	18.2	22	3.2	**0.85 ± 0.06**	**10**	**1.4 × 10**^-7^***

*POSH*^*RNAi*^*/GMR-Gal4*	13.3	15	0.9	-	-	-

*Rack1*^*RNAi*^*/GMR-Gal4*	7.1	14	6.6 × 10^-4^	0.94 ± 0.06	11	0.13

*robo*^*RNAi*^*/GMR-Gal4*	**25.0**	**20**	7.8 **	0.92 ± 0.04	6	0.14

*SP2353*^*RNAi*^*/GMR-Gal4*	16.7	12	2.4	**0.94 ± 0.05**		0.014*

*Nrk*^*RNAi*^*/GMR-Gal4*	-	-	-	**0.82 ± 0.05**	**14**	**3.8 × 10**^-11^***

*CG34400*^*RNAi*^*/GMR-Gal4*	-	-	-	**0.81 ± 0.05**	**17**	**2.0 × 10**^-9^***

*Grh*^*RNAi*^*/GMR-Gal4*	-	-	-	**0.86 ± 0.04**	**6**	**1.1 × 10**^-4^***

*vimar*^*RNAi*^*/GMR-Gal4*	9.5	21	1.0	**0.76 ± 0.05**	**13**	**2.2 × 10**^-15^***

*Pgk*^*RNAi*^*/GMR-Gal4*	-	-	-	**0.77 ± 0.04**	**18**	**4.2 × 10**^-6^***

all mutant alleles were obtained from BDSC, all RNAi mutants - from VDRC, ^1 ^- from DGRC

^2 ^Vienna stock number v21995

^3 ^Vienna stock number v101588

Mutations in many of the DGC-interacting genes cause visual system defects; therefore, we analyzed the expression of these genes in the *Drosophila *nervous system via examination of published data and available GFP expression lines. *Lis1 *mRNA was found in the brain hemispheres and eye imaginal discs of 3^rd ^instar larvae [36]. Mbl is also expressed in larval eye discs and is required for photoreceptor differentiation and *mbl *deficiency results in shortened rhabdomeres [37]. In addition, expression in the central and peripheral nervous system has been shown for Grh [38], Nrk [39], Vimar [40] and Robo [41]. Interestingly, Robo is a transmembrane receptor for the extracellular matrix protein Slit, and previous reports showed that *robo *mutation results in improper axon crossing in the embryo and defects in compartmentalization of visual centers in the larval and adult brain [42-44].

We used modENCODE temporal expression data [45] to determine the expression of *chif, CG34400 *and *SP2353*. Expression of *SP2353 *is enriched in the adult brain and thoracic-abdominal ganglion. *CG34400 *and *chif *are expressed during development and adulthood and a previous report showed that *chif *mutants have a rough eye phenotype [46].

We also used GFP trap lines to recognize the expression pattern for Cam and Capt in the larval brain. We identified that Capt is expressed ubiquitously in the central and ventral brain and its expression is enriched in optic lobes and eye discs (Figure 2L-M). Cam has a more defined expression pattern in the neuropile and central brain and is also enhanced in optic lobes and eye discs (Figure 2N-O). Capt and Cam are expressed in R cells of the eye disc (Figure 2M,O) and enriched in the area where R1-6 axons terminate, similarly to Dys and Dg suggesting that they may act in the same cell types (Figure 1A,C). Since many of the proteins that interact with the DGC in muscle are expressed in the nervous system and have comparable phenotypes in the visual system to *Dys *and *Dg *mutants, we determined if they genetically interact with *Dys *and *Dg *in this tissue as well.

### Search for Dys and/or Dg interacting partners in photoreceptor axon pathfinding

**Figure 3:**
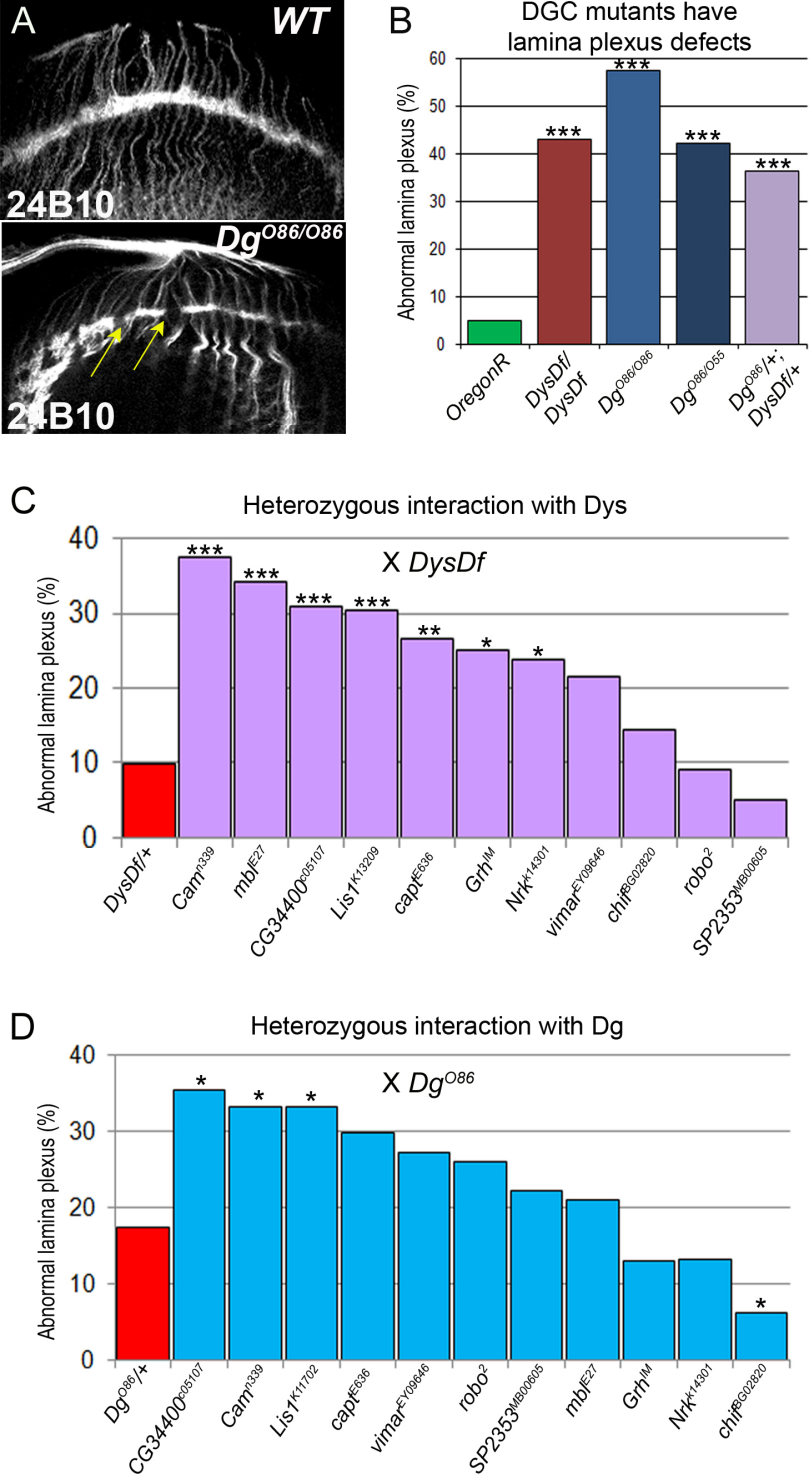
**Genetic interactors were found to cause lamina plexus defects with Dys and Dg**. (A) Wild type properly formed lamina plexus and defects found in Dg (and Dys) mutants showing gaps in the axon projections (arrows). 24B10 antibody was used to visualize photoreceptor axons. (B) Dys and Dg homozygous mutants have a significantly higher percentage of these defects from control and genetically interact. (C) Genetic interactions found with *Dys*, and with *Dg *(D). Statistics were determined using the χ^2^-test with Yate's correction where ***p ≤ 0.001, **p ≤ 0.01, *p ≤ 0.05.

First we looked at photoreceptor axon pathfinding to assess the genetic interaction of *Dys *and *Dg *with components shown to be required in visual system neurons. For genetic analysis we used loss-of-function mutants for *Dys *and *Dg *that in homozygous and heterozygous state demonstrate breaks in the lamina plexus (Figure 3A-B). Since reduction of one copy of *Dys *or *Dg *has a mild phenotype (Figure 3C-D), they can be used for a transheterozygous genetic interaction analysis in photoreceptor cell projections. Deleting one copy of an interacting gene in the heterozygous *Dg *or *Dys *mutant background will increase the frequency of abnormal lamina plexuses if there is a genetic interaction. Seven genes were found to increase the appearance of abnormal lamina plexuses in a *Dys *heterozygous background and three in a *Dg *heterozygous background (Figure 3C-D, Table 2). To avoid an additive effect, we tested the found interactors for a dominant phenotype. Three genes, *Lis1, CG34400 *and *Grh *have abnormal lamina plexuses while reduced by one copy (37.5%, 14.3% and 10.0% respectively) and were therefore not considered to interac. In summary, in this screen we have identified Nrk - a protein with tyrosine kinase activity, Cam - a main player in calcium-mediated signaling, Mbl - a DNA binding protein implicated in mRNA splicing, and Capt - a factor required to prevent actin filament polymerization.

**Table 2:** Genetic interaction with *Dys *and *Dg *in photoreceptor axon path finding

Gene name	Defective lamina plexuses (%)	n, analyzed optic lobes	**χ**^**2**^**-value**	Defective lamina plexuses (%)	n, analyzed optic lobes	**χ**^**2**^**-value**	Defective lamina plexuses (%)	n, analyzed optic lobes	**χ**^**2**^**-value**
	***DysDf *x**	***DysDf *x**	***DysDf *x**	***Dg***^***O86 ***^**x**	***Dg***^***O86 ***^**x**	***Dg***^***O86 ***^**x**	***OregonR *x**	***OregonR *x**	***OregonR *x**

*OregonR*	9.8	61	1.1^°^	17.5	40	6.0^° *^	4.9	41	-

*DysDf*	**42.9**	**49**	**28.8**^° ^***	**36.4**	**55**	**22.5**^° ^***	9.8	61	1.1^°^

*Dg[O86]*	**36.4**	**55**	**22.5**^° ^***	**57.5**	**40**	**42.7**^° ^***	17.5	40	6.0^° *^

*Dg[O55]*	-	-	-	**42.1**	**19**	**27.9**^° ^***	-	-	-

*Cam[n339]*	**37.5**	**48**	15.0 ***	**33.3**	**39**	4.3 *	0.0	9	3.0

*Capt[E636]*	**26.7**	**45**	6.9 **	29.8	47	2.7	2.9	35	0.1

*CG34400[c05107]*	**30.9**	**42**	9.9 **	**35.5**	**31**	5.4 *	14.3	35	3.7

*CG7845[EMS-Mod4]*^*1*^	11.1	9	4.0 × 10^-3^	**5.6**	**18**	5.2 *	-	-	-

*chif[BG02820]*	14.3	14	0.5	**6.3**	**16**	4.4 *	-	-	-

*Fhos[A055]*	19.0	21	2.3	8.3	12	2.6	-	-	-

*Fkbp13[P962]*	21.4	14	3.6	11.1	9	1.0	-	-	-

*Grh[IM]*	**25.0**	**24**	5.8 *	13.0	23	0.4	10.0	20	1.2

*Lis1[k11702]*	17.8	28	1.8	31.2	16	3.3	7.7	13	0.3

*Lis1[k13209]*^*2*^	**30.3**	**33**	9.5**	**33.3**	**12**	4.3*	**37.5**	**16**	23.7***

*Lis1[k13209]*	**33.3**	**12**	11.8***	11.1	9	1.0	**28.6**	**14**	15.5***

*mbl[E27]*	**34.3**	**35**	12.5 ***	21.0	38	0.2	0.0	7	3.0

*nAcRα-30D[EY13897]*	**0.0**	**9**	7.9 *	12.5	8	0.5	-	-	-

*Nrk[k14301]*^*2*^	**23.7**	**38**	4.9 *	13.3	15	0.3	0.0	7	3.0

*POSH[k15815]*^*2*^	9.1	22	5.0 × 10^-3^	21.7	23	0.3	-	-	-

*Rack1[EY00128]*^*2*^	**0.0**	**13**	7.9 *	21.7	23	0.3	-	-	-

robo[2]	9.1	11	4.0 × 10^-3^	26.1	23	1.3	-	-	-

*SP2353[MB00605]*	5.0	20	1.0	22.2	18	0.3	-	-	-

*vimar[EY09646]*	21.4	42	3.6	27.3	22	1.7	-	-	-

all mutant alleles were obtained from BDSC, all RNAi mutants - from VDRC, ^1 ^- described previously [70], ^2 ^- from DGRC

^° ^- compares to *Oregon R*

### Genetic interaction with Dys and Dg in controlling rhabdomere elongation

**Figure 4:**
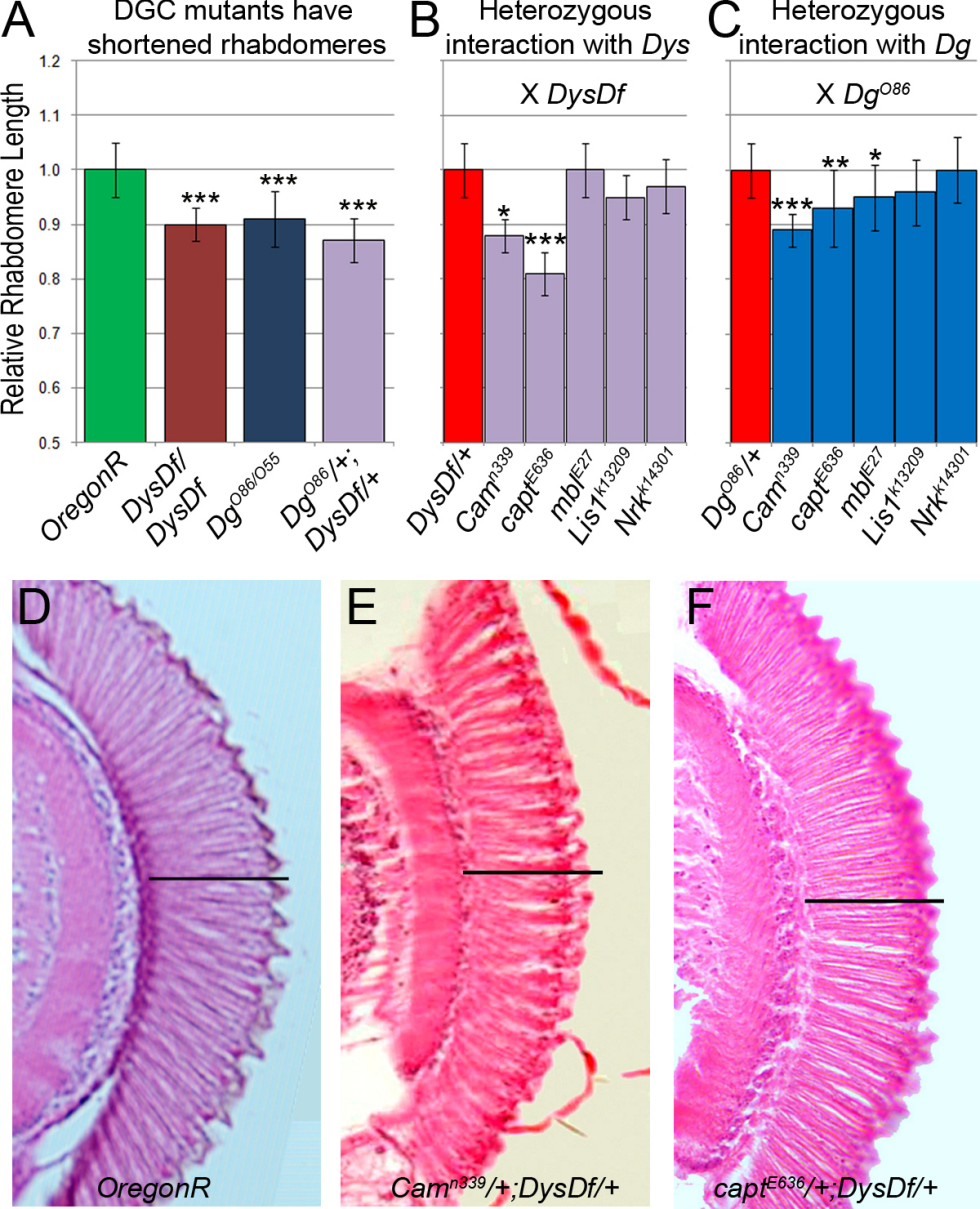
**Genetic interactors were found to cause shortened rhabdomeres with Dys and Dg**. (A) *Dys *and *Dg *homozygous mutants have significantly shorter rhabdomeres in the developed adult eye and interact in this process. Measurements have been normalized to control. (B) Genetic interactions found with *Dys*, and with *Dg *normalized to *Dys/+ *or *Dg/+ *appropriately (C). Rhabdomere examples from *OregonR *(WT,D), *Cam*^*n339*^*/+;DysDf/+ *(E) and *capt*^*E636*^*/+;DysDf/+ *(F). Scale bare represents *wt *rhabdomere length. Statistics were determined using a one-tailed Student's t-test where ***p ≤ 0.001, **p ≤ 0.01, *p ≤ 0.05.

To complement the data derived from analysis of 3^rd ^instar larval brains, we have performed a separate assay to evaluate the genetic interaction with the DGC in the process of rhabdomere (photoreceptor cell) elongation. First, we analyzed histological sections of *Drosophila *adult eyes and showed that *Dys *and *Dg *loss-of-function mutants also have shortened rhabdomeres, and reduction by one copy of both *Dys *and *Dg *results in a genetic interaction (Figure 4A). Next we found that *Cam *and *capt *genetically interact with *Dys *(Figure 4B, E-F, Table 3) and *Cam*, *capt *and *mbl *with *Dg *(Figure 4C, Table 3) displaying significantly shortened rhabdomeres. None of the tested mutants in a heterozygous state showed a significant phenotype (1.04 ± 0.05, 0.97 ± 0.02, 1.03 ± 0.06, 1.00 ± 0.02 and 1.04 ± 0.04 for *capt*, *Nrk*, *mbl, Lis1 *and *Cam *respectively when normalized to *wt *control).

**Table 3:** Genetic interaction with *Dys *and *Dg *in rhabdomere elongation

Gene name	Relative rhabdomere length	n, analyzed eyes	p-value	Relative rhabdomere length	n, analyzed eyes	p-value
	***DysDf *x**	***DysDf *x**	***DysDf *x**	***Dg***^***O86***^	***Dg***^***O86***^	***Dg***^***O86***^

*OregonR*	1.00 ± 0.05	6	-	1.00 ± 0.05	20	-

*DysDf*	**0.89 ± 0.03**	**15**	**4.6 × 10**^**-5**^	**0.80 ± 0.04**	**12**	**3.5 × 10**^**-10**^

*Dg[O86]*	**0.85 ± 0.04**	**12**	**4.0 × 10**^**-5**^	-	-	-

*Dg[O55]*	-	-	-	**0.83 ± 0.05**	**15**	**3.7 × 10**^**-9**^

*Cam[n339]*	**0.88 ± 0.03**	**3**	**1.4 × 10**^**-2**^	**0.89 ± 0.03**	**6**	**3.7 × 10**^**-4**^

*Capt[E636]*	**0.81 ± 0.04**	**6**	**5.0 × 10**^**-5**^	**0.93 ± 0.07**	**12**	**1.0 × 10**^**-2**^

*Mbl[E27]*	1.00 ± 0.05	6	0.5	**0.95 ± 0.06**	**14**	**2.8 × 10**^**-2**^

*Nrk[k14301]*	0.97 ± 0.05	12	0.2	1.00 ± 0.06	12	0.5

*Lis1[k13209]*	0.95 ± 0.04	7	0.087	0.96 ± 0.06	13	0.084

Here we have found genes that interact with *Dys *and/or *Dg *in axon pathfinding and in the process of photoreceptor cell elongation. Shortened rhabdomeres can result from actin cytoskeletal defects, but also from improper photoreceptor cell fate specification, retinal degeneration or abnormal innervations. Dynamic changes in actin filaments provide cell shape and control photoreceptor cell differentiation in developing *Drosophila *pupae. Therefore disruption of these processes may also affect the rhabdomere elongation in the *Drosophila *adult eye.

### The DGC coordinates actin cytoskeleton remodeling

**Figure 5:**
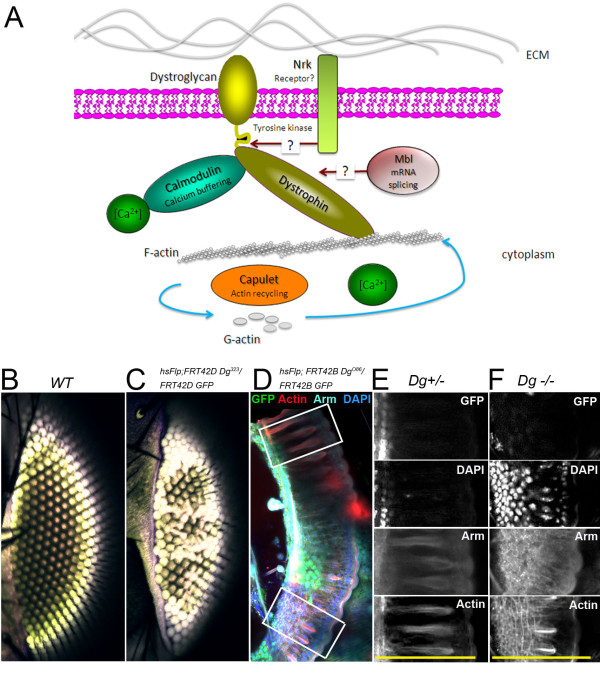
**The DGC coordinates actin cytoskeleton remodeling**. (A) Scheme showing a cell with the DGC and found Dys/Dg-interacting components. Autofluorescence image shows a regular ommatidia pattern (B) which is disrupted by *Dg*^*323 *^clone induction (C). (D) *Dg*^*O86 *^mutant clones (marked by the absence of nuclear GFP) in a *Drosophila *pupal eye stained with anti-phalloidin (Actin) and the nuclear marker DAPI. Rectangles indicate *wt *(upper) and mutant (lower) areas. An enlarged view of *wt *(E) and mutant (F) zones (indicated with rectangles in D) with GFP, DAPI, Arm and Actin cell markers in different channels. Dg^-/- ^cells (GFP negative) have disorganized nuclei, improper Arm and Actin localization and shorter rhabdomeres when compared to sister *wt *cells (GFP positive). Yellow bars in lower panel indicate normal rhabdomere length.

In our screen we identified Cam, Capt and Mbl as DGC-interacting partners that have roles in actin dynamics (Figure 5A), which implies that the phenotypes observed in the DGC mutants might at least partially result from improper actin cytoskeleton organization. To explore this hypothesis we generated clones in developing *Drosophila *eye discs homozygous for *Dg *loss-of-function alleles using the FLP/FRT system. These clones resulted in irregular ommatidia in adult animals (Figure 5B-C). For detailed characterization of this phenotype we performed immunohistochemical analysis of clonal pupal retinas using multiple cell markers including actin (Figure 5D-F). First we noticed that clones (marked by absence of GFP) have shortened ommatidia (Figure 5D-F, yellow bars). Further analysis revealed that there was also disrupted layering of nuclei. DAPI staining made clear that nuclear migration during development does not occur properly, resulting in disorganized layers (Figure 5 E-F). We also observed a different pattern of β-catenin (Arm) in clonal areas compared to wild type (Figure 5E-F) suggesting that during development cells lacking Dg cannot change their shape and elongate properly. In addition, irregular Actin was seen in *Dg *mutant clones indicating DGC involvement in actin dynamics in photoreceptor cells. This result suggests that the DGC cell autonomously coordinates actin cytoskeleton remodeling, the process required for proper photoreceptor cell growth and elongation, as well as axon migration during *Drosophila *eye development; it further indicates that photoreceptor axon pathfinding and R-cell elongation defects observed in DGC mutants might be a result of improper actin reorganization during development.

## Discussion

The roles that Dys and Dg play in disease have been apparent for some time since their disruption or misregulation has been closely linked to various MDs. Dg depletion results in CMD-like brain malformations associated with layering defects and aberrant neuron migration [34]. These defects arise due to extracellular matrix protein affinity problems that influence neuronal communication and result in learning and memory defects. Similar to brain layer formation, the migration of R1-R6 growth cones into the lamina occurs in a similar manner where glia cells that migrate from progenitor regions into the lamina provide a termination cue to innervating axons. In *Drosophila *Dys and Dg are expressed in the CNS, PNS and visual system and both proteins are required for proper photoreceptor axon guidance and rhabdomere elongation [24,33]. In this work we identified novel components implicated in the process of eye-neuron development. Moreover, we found that Nrk, Mbl, Cam and Capt genetically interact with Dys and/or Dg in visual system establishment.

The proteins Mbl, Capt, Cam, Robo, Lis1 and Nrk have been shown previously to be associated with the nervous system, and now we have additionally found that mutations in *chif, SP2353, CG34400 *and *vimar *cause abnormal photoreceptor axon pathfinding and/or differentiation phenotypes. Lis1 has been shown to bind microtubules in the growth cone [47], and the human Lis1 homologue is important for neuronal migration and when mutated causes Lissencephaly, a severe neuronal migration defect characterized by a smooth cerebral surface, mental retardation and seizures [48]. Now we have found that *Lis1*^*RNAi*^*/GMR-Gal4 *mutants have abnormally formed lamina plexuses, shortened rhabdomeres, and retinal vacuoles. Chif has been shown to regulate gene expression during egg shell development and is related to a DNA replication protein in yeast [46]. The human ortholog for SP2353 (AGRN) is involved in congenital MD development [49,50]. *Drosophila *SP2353 is a novel agrin-like protein that contains Laminin G domains, which makes it a potential new extracellular binding partner for Dg. *CG34400 *encodes for a protein homologues to human DFNB31 (Deafness, autosomal recessive 31) that causes congenital hearing impairment in DFNB31 deficient people and mouse whirlin, that causes deafness in the whirler mouse [51]. Hearing loss has been as well demonstrated in association with various forms of muscular dystrophy [51]. Vimar has been shown to regulate mitochondrial function via an increase in citrate synthase activity [52].

*Mbl *is a *Drosophila *homologue of the human gene MBNL1. Mutations of this gene cause myotonic dystrophy and are associated with the RNA toxicity of CUG expansion diseases protein [53]. Here we show that Mbl deficiency results in similar phenotypes to Dys and Dg loss of function, and to specifically interact with *Dys *in axon projections which is in accord with the *Dys *specific interaction seen in muscle [35]. Dys has multiple isoforms, and the variability of DMD patients to have mental impairment has been linked in part to small Dys isoform mutations, which leads to speculation that Dys is a target for Mbl mediated splicing.

Interestingly, Mbl isoforms have been demonstrated to regulate splicing of α-actinin [54], which belongs to the spectrin gene superfamily that also includes dystrophins. α-actinin and Capt, the *Drosophila *homologue of Cyclase-associated protein (CAP) are actin-binding proteins in the growth cone. Capt was first identified in yeast and is highly conserved throughout eukaryotic evolution [55]. The main known function of Capt is to act in the process of actin recycling by working in conjunction with Actin Depolymerization Factor (ADF a.k.a. Cofilin) to help displace Cofilin from G-actin during depolymerization [56,57]. It has already been reported that ADF/Cofilin has a role in retinal elongation [58]. The actin cytoskeleton is a major internal structure that defines the morphology of neurons, and Capt has already been shown to be required to maintain PNS neuronal dendrite homeostasis in *Drosophila *via kinesin-mediated transport [59]. Additionally, Capt has been found to lead to excessive actin filament polymerization in the eye disc and to cause premature differentiation of photoreceptors [60]. The rate of axon projection is much slower than the rate of microtubule polymerization during axonal growth [61], implying that depolymerization/polymerization of actin is important during pathfinding. We have also shown that Capt interacts with Dys and is necessary for proper projection of photoreceptor axons in the developing brain, and when absent, eyes develop with abnormal rhabdomeres. Furthermore, we have demonstrated that *capt*^*RNAi *^mutants exhibit overgrowth of photoreceptor axons, and we believe a possible explanation for this is improper turnover of actin (Figure 5A).

Importantly, proteins that can be regulated by Ca^2+ ^to organize actin filament bundles and to promote filament turnover include α-actinin and (ADF)/Cofilin, respectively [62-64]. Cam functions as an intracellular Ca^2+ ^sensor, and when Ca^2+^-Cam was selectively disrupted in a subset of neurons in *Drosophila *embryos, stalls in axon extension and errors in growth cone guidance resulted [65]. Actin turnover is highly regulated by Ca^2+ ^levels, and many proteins are Ca^2+^-mediated to regulate motility and axon guidance. Our results and those from prior studies suggest that Cam is a major functional player of Ca^2+ ^regulation in growth cones. Since we show here that mutations in *Cam *and *capt *have similar phenotypes in photoreceptor axon pathfinding and rhabdomere development, we postulate that actin dynamics is the link between these two proteins and the phenotypes described here. Due to the importance of Cam for actin dynamics, its interaction with both Dg and Dys suggests that the DGC coordinates the actin cytoskeleton in the developing eye.

The last gene that we have identified in this work is Nrk. Recently various kinases, channels and other enzymes have been shown to associate with the DGC, although only a few of these interactions have been confirmed *in vivo *[66,67]. Since Nrk is a component found to interact with *Dys *in photoreceptor axon pathfinding, it is most likely that it functions as a receptor to sense guidance cues rather than as a molecule affecting actin cytoskeletal rearrangement. Our data here hint that Dg and Nrk could be two receptors integral to transferring signals important for neuronal layering.

## Conclusions

Dynamic rearrangement of the actin cytoskeleton is crucial for the central and peripheral nervous system establishment, which depends on proper neuron migration and differentiation. This process requires not only the cell autonomous regulation of neuron motility, but also the interaction between the migrating cell and its underlying substrate. This interaction is often dependent on the signaling transduced via the ECM. The DGC and other factors are believed to be mediators of actin dynamics in growing axons and during neuronal cell morphogenesis, and our study found components that interact with Dys and/or Dg in both of these activities (Figure 5A). Additionally, disruption in gene expression of these components results in the same phenotypes seen with *Dys *and *Dg *mutants in the developing and adult eye. Our data allows us to conclude that the DGC is involved in signaling to cause cytoskeletal rearrangement and actin turnover in growth cones (Figure 5A). Since many cases of muscular dystrophies are associated with mental retardation, we believe that it is important to understand the role of the DGC in axon migration because understanding of this process could aid in finding an adequate therapy for this aspect of the disease's physiology. Since the human brain continues to develop well after gestation, and evidence shows that nerves maintain plasticity throughout an individual's lifespan, therapies could be devised that reverse these defects after birth.

## Methods

### Fly Strains and Genetics

Fly stocks were maintained at 25°C on a standard cornmeal-agar diet. Fly strains used in this study are: loss of function mutants *DysDf, Dg*^*O86*^*, Dg*^*O55 *^[68], *Dg*^*323 *^[69]*GMR-Gal4 *and *OregonR *(wild type). Lines carrying screened mutations include the following alleles: *Cam*^*n339*^, *Grh*^*IM*^, *mbl*^*E27*^*, CG34400*^*c05107*^*, Capt*^*E636*^*, Nrk*^*k14301*^*, Fkbp13*^*P962*^*, vimar*^*EY09646*^*, Fhos*^*A055*^*, Lis1*^*k11702*^*, Lis1*^*k13209*^*, FRT42D-Lis1*^*k13209 *^(Kyoto DGRC)*, chif*^*BG02820*^*, CG7845*^*EMS-MOD4 *^[70], *POSH*^*k15815*^*, robo*^*2*^*, SP2353*^*MB00605*^*, nAcRα-30D*^*Ey13897*^*, Rack1*^*EY00128*^. Unless otherwise stated, lines were obtained from BDRC. *RNAi *lines were obtained from the VDRC and line numbers are as follows: *Cam (v28242), capt (v21995 and v101588), CG34400 (v28945), Fkbp13 (v12863), Lis1 (v106777), mbl (v28731), Nrk (v36282), Rack1 (v104470), robo (v4329), vimar (v21686), POSH (v26655) *and *Grh (v33679)*. To determine protein expression of Capt and Cam we obtained GFP protein trap lines from the FlyTrap project [71] that generates a fused GFP protein (*Cam*^*P00695*^) or GFP expression is controlled by enhancer elements (*capt*^*YB0070*^).

Homozygous lethal lines were balanced over the *CyO *balancer chromosome marked with Kruppel-GFP to make it possible to determine the genotypes of larvae. Third chromosome alleles were balanced with the *TM6,Tb *balancer chromosome which results in shorter and thicker larvae allowing for its detection. Non-GFP and non-Tb progeny (F1) were collected from crosses at the L3 larval stage of development for axon path-finding analysis and as adult flies for retina length determination.

*Dg *mutant clonal cells were generated by crossing females of genotype *hsFlp; FRT 42B GFP/CyO *with males of genotype *FRT 42B Dg*^*O86*^*/CyO*. Vials were exposed to 2 hrs of 37°C heat shocks per day starting 1 day AEL until pupae formation. Dissection of eyes was done approximately 70 hrs APF.

### Immunohistochemistry

Dissections were done in PBS, fixed in 4% formaldehyde and antibodies were applied as described previously [24]. The following antibodies were used: mouse anti-24B10 (1:50, Development Studies Hybridoma Bank), rabbit anti-Dg [69] (1:1000), anti-Dp186 and anti-DysCO_2_H [28] (1:600), anti-Arm, Alexa 488 and 568 goat anti-mouse, Alexa 488 goat anti-rabbit (1:500, Molecular probes) and Alexa 568 conjugated phalloidin (1:40, Invitrogen). DAPI was used to visualize nuclei. Samples were mounted on slides in 70% glycerol, 2% NPG, 1X PBS and analyzed using a confocal microscopes (Leica TCS SP5, Zeiss Axio Imager).

### Histology

For analysis of eye and head morphology, 10 μm paraffin-embedded sections were cut of fly heads. In order to prepare *Drosophila *sections, fly heads were immobilized in collars in the required orientation and fixed in Carnoy fixative solution (6:3:1 Ethanol:Chloroform:Acetic acid) at 4°C overnight. Tissue dehydration and embedding in paraffin was performed as described previously [72]. Histological sections were prepared using a Hyrax M25 (Zeiss) microtome and stained with hematoxylin and eosin. All chemicals for these procedures were obtained from Sigma Aldrich. Analysis was done using a light microscope (Zeiss). To prepare *Drosophila *adult brain cryosections the protocol adapted from [72] was used. First flies were located in collars and immediately frozen in TissueTek^® ^O.C.T. (Tissue-Tek) at ≈ -40°C. Then frozen heads were sectioned on a cryo-microtom Leica CM3050S with a section thickness of 10 μm. Fixation was carried out in 4% formaldehyde (Polyscience, Inc.) for 10 min at room temperature.

### Data Analysis

The percentage of larval brain lobes with abnormalities in the lamina plexus were quantified as the percentage of defective lobes divided by the total lobes examined. Adult ommatidia lengths were measured and normalized to the appropriate control.

### Statistics

Statistical analysis of abnormal lamina plexus formation was done using a one-tailed χ^2 ^test. Statistical analysis of ommatidia length was done using a one-tailed Student's t-test where error bars represent the average deviation. For transheterozygous interation of screened genes with *Dys *and *Dg *comparisons were made to *Dys/+ *or *Dg/+ *as appropriate.

## Abbreviations

DGC: Dystrophin Glycoprotein Complex; Dys: Dystrophin; Dg: Dystroglycan; MD: muscular Dystrophy

## Authors' contributions

AKM, MMK and VMR carried out all immunohistochemistry and histological experiments. AKM, MMK and HRS participated in the design of the study and drafted the manuscript. All authors read and approved the final manuscript.
